# Olfactory Preferences of *Sitophilus zeamais* to Cereal- and Legume-Based Pasta

**DOI:** 10.3390/insects15030167

**Published:** 2024-02-29

**Authors:** Pasquale Trematerra, Giacinto Salvatore Germinara, Marco Colacci

**Affiliations:** 1 Department of Agricultural, Environmental and Food Sciences, University of Molise, Via de Sanctis, I-86100 Campobasso, Italy; trema@unimol.it; 2 Department of Agricultural Sciences, Food, Natural Resources and Engineering, University of Foggia, Via Napoli 25, I-71122 Foggia, Italy

**Keywords:** maize weevil, olfactory arena tests, pasta preferences

## Abstract

**Simple Summary:**

Several insects can infest alimentary pasta. Since the olfactory sense in insects plays an important role in locating food sources, the aim of this work was to investigate the responses of adult maize weevils, *Sitophilus zeamais*, to five commercially available categories of macaroni pasta made from cereals and/or legumes in an olfactometric arena. Maize weevil adults were more attracted to the pasta made of 100% of *Triticum durum*; the attractiveness strongly decreased when combining *T. durum* with *Cicer arietimun* or *Lens esculenta.* A preferential orientation of adult insects to pasta made of chickpeas or lentils was not observed. These results confirm the importance of volatile compounds in insect orientation towards suitable foods and oviposition substrates and provide useful practical information for the safer prevention of maize weevil pasta infestations.

**Abstract:**

We compared the attractiveness of five commercially available Italian macaroni pastas of different shapes (penne, casarecce, and fusilli) made from cereals and/or legumes [100% *Triticum durum*; 100% *Cicer arietinum*; 100% *Lens esculenta*; 50% *Triticum durum* + 50% *Cicer arietinum*; 60% *Triticum durum* + 40% *Lens esculenta*] to adults of *Sitophilus zeamais* (L.). A multiple-choice walking bioassay showed that *S. zeamais* adults were more attracted to cereal than legume pastas. The modified Flit-Track M^2^ trap devices baited with pasta made with 100% *T. durum* captured an average of 61.4% of the adults released into the olfactometric arena after 7 days. Of the insects tested, pasta made with 100% *C. arietinum* trapped 3.8%, pasta made with 100% *L. esculenta* trapped 2.7%, pasta made with 50% *T. durum* + 50% *C. arietinum* trapped 4.3%, and pasta made with 60% *T. durum* + 40% *L. esculenta* trapped 4.2%. When individually compared, 79.6% of *S. zeamais* adults chose the *Triticum durum* pasta. Orientation to 100% *Cicer* pasta or 100% *Lens* pasta was not observed. In the choice test, only 37% and 25% were attracted to *Triticum* and *Cicer* pastas or *Triticum* and *Lens* pasta, respectively. Our results confirm that the low attractiveness of legume pasta is mainly due to the lack of attractant stimuli rather than the emission of repellent compounds. From a practical perspective, it is also interesting to note how mixed pasta decreases the risk of *S. zeamais* infestation.

## 1. Introduction

As is already known, alimentary pasta can be infested by several insects (Lepidoptera and Coleoptera), leading to high levels of economic damage. Infestations can occur from product production to use (e.g., storage processes in industries, shipment, warehouses, general stores and retail shops, and the consumer’s house) [1,2,3,4,5,6,7].

Among other insects, maize weevils of the *Sitophilus* species are considered to be one of the most destructive primary pests of stored cereals [8]. They prefer to feed on cereal products, although they can feed on split peas and various types of pasta [6]. In commercial pasta packages, adult weevils can penetrate by enlarging the vent micro-holes (from which the odour that attracts the insect escapes), either through openings actively made by themselves, or through pre-existing mechanical damage [3,6,9].

With a view to providing the consumer with a safer and better-quality product, and to avoiding insect infestations, which would compromise the brand image in the eyes of consumers, resistant and sealed packaging has been proposed [10,11,12,13,14,15,16].

In this context, the market also demands new types of pasta that are higher in protein, rich in vegetable fibres and certified as gluten-free or non-GMO. For these reasons the pasta industry has produced and marketed products made, for example, using various flours derived from legumes.

In the present work, we continue our research related to the relationships between *Sitophilus* spp. and different types of pasta [4,6]. Because the olfactory sense in *S. zeamais* plays an important role in the infestation of a food in response to food odours, we investigated, in an olfactometric arena, the responses of adult maize weevils to five categories of macaroni pasta (100% *Triticum durum* pasta; 100% *Cicer arietinum* pasta; 100% *Lens esculenta* pasta; 50% *Triticum durum* + 50% *Cicer arietinum* pasta; 60% *Triticum durum* + 40% *Lens esculenta* pasta) in order to evaluate the effect of odours emitted by the different pasta products on insect behaviour.

## 2. Materials and Methods

### 2.1. Insects

Unsexed 1- to 2-week-old *S. zeamais* adults, from a wild population found and reared on barley with no history of exposure to insecticides were used in behavioural bioassay tests. The maize weevil colony was reared in a climatic chamber at 28 ± 2 °C and 70 ± 5% RH, with an L12:D12 photoperiod. 

### 2.2. Pasta Types

Five commercially available Italian macaroni pasta brands of different shapes (penne, casarecce, and fusilli) (samples A–E) made of cereal- and/or legume-based [100% *T. durum* (sample A); 100% *C. arietinum* (sample B); 100% *L. esculenta* (sample C); 50% *T. durum* + 50% *C. arietinum* (sample D); 60% *T. durum* + 40% *L. esculenta* (sample E)] were tested. The nutritional information (average values) for 100 g of each macaroni pasta is reported in Table 1.

### 2.3. Multiple-Choice Behavioural Bioassays

The multiple-choice behavioural bioassays were carried out in cylindrical Plexiglas arenas (80 cm diameter × 40 cm high). Each arena contained 6 modified Flit-Track M^2^ trap devices (Trécé Incorporated, Adair, OK, USA); 5 trap devices baited with 10 g of the different pasta types (samples A–E) and one left empty used as a control (sample F). 

Following the experimental design reported in Trematerra et al. (2021) [6], 12 replications (100 *S. zeamais* adults released at the centre of the arena) were performed, using a total of 1200 insects used once. The number of trapped insects was checked 7 days after their introduction into the arena. After each replication, the positions of the trap devices were randomized and their contents renewed. The tests were carried out in incubators, under controlled conditions: 28 ± 2 °C, 70 ± 5% RH.

### 2.4. Two-Choice Behavioural Bioassays

The attractiveness of the cereal and legume pastas was compared using the cylindrical olfactometer arena, the modified Flit-Track M^2^ trap devices and the methodology described above. 

The numbers of trapped and not trapped (free in the arena) insects were checked 7 days after their release into the arena. The following pairwise comparisons were performed (first choice vs. unbaited control): 100% *T. durum* pasta vs. control (samples A vs. F); 100% *C. arietinum* pasta vs. control (samples B vs. F); 100% *L. esculenta* pasta vs. control (samples C vs. F); 50% *T. durum* + 50% *C. arietinum* pasta vs. control (samples D vs. F); 60% *T. durum* + 40% *L. esculenta* pasta vs. control (samples E vs. F). 

For each experiment, 5 replications were performed. In each trial, a response index (RI) was calculated following Trematerra et al. (2021) [6].

### 2.5. Data Analysis

Friedman two-way ANOVA by ranks was used to analyse the numbers of insects found in the different modified Flit-Track M^2^ trap devices for the multiple-choice behavioural bioassays. In the case of significance (*p* < 0.05), the Wilcoxon signed ranks test was used for separation of means.

Student’s *t*-test was used to compare the mean numbers of insects found in the two choices behavioural bioassays. One-way ANOVA followed by the post hoc Tukey HSD test was used to evaluate the different RIs.

Statistical analyses were carried out using the statistical software SPSS version 13.0 (SPSS Inc., Chicago, IL, USA).

## 3. Results

### 3.1. Multiple-Choice Behavioural Bioassays

**Figure 1:**
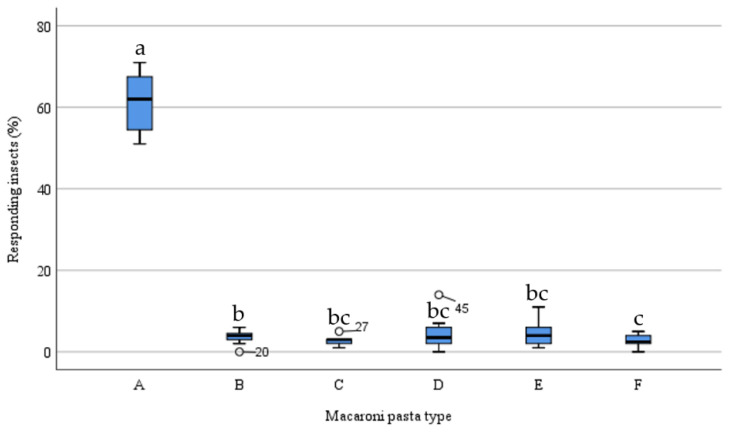
Olfactory responses of *S. zeamais* adults to 100% *T. durum* pasta (A), 100% *C. arietinum* pasta (B), 100% *L. esculenta* pasta (C), 50% *T. durum* + 50% *C. arietinum* pasta (D), 60% *T. durum* + 40% *L. esculenta* pasta (E), and the control trap device (F) in the multiple-choice bioassay. Each box plot indicates the median and its range of dispersion (lower and upper quartiles). Dots = outliers. Above each box plot, different letters indicate significant differences (Wilcoxon test, *p* < 0.05).

Figure 1 shows the olfactometric responses of *S. zeamais* adults to different pasta types. Significant differences (χ^2^ = 32.181, *df* = 5, *p* < 0.001) in adults captured by the modified trap devices containing different pastas were recorded in the multiple-choice behavioural bioassays 

The trap devices baited with pasta made of 100% *T. durum* (A) captured an average of 61.4% of the adults released into the olfactometric arena. The number of insects attracted by 100% *T. durum* pasta was significantly higher (Wilcoxon test, *p* < 0.05) than those attracted by the other four pasta types and the control trap device: 100% *C. arietinum* (B) trapped 3.8%, 100% *L. esculenta* (C) trapped 2.7%, 50% *T. durum* + 50% *C. arietinum* (D) trapped 4.3%, 60% *T. durum* + 40% *L. esculenta* (E) trapped 4.2%, and the control trap device (F) trapped 2.7%.

Samples C, D, and E showed no statistical differences (Wilcoxon test, *p* > 0.05) with the control trap device, instead the number of insects attracted by sample B was significantly higher.

### 3.2. Two-Choice Behavioural Bioassays

Table 2 shows the results of the two-choice behavioural bioassays. Significant differences were found among the RIs to different stimuli tested (ANOVA: F = 49.200, df = 4, *p* < 0.001). 

The RIs induced by *T. durum*, *T. durum* + *C. arietinum*, and *T. durum* + *L. esculenta* pastas were positive and significant (*p* < 0.05, Student’s *t*-test) whereas those of the *C. arietinum* and *L. esculenta* pastas were not significant, respectively, indicating actual attraction and no preferential orientation of adult maize weevils towards different test odours. Moreover, of the attractant stimuli, the mean RIs of *T. durum* pasta (76.6 ± 4.3) were significantly higher (*p* < 0.05, Tukey HSD post hoc test) than those of pastas made of *T. durum* + *C. arietinum* (28.6 ± 7.7) and *T. durum* + *L. esculenta* (11.6 ± 1.1).

## 4. Discussion

As mentioned earlier, pasta can be infested by different insect species in various parts of the production and distribution chains. Among the most common and dangerous species are those of the genus *Sitophilus*. The susceptibility of various pasta types to insect infestation has been investigated in previous studies providing practical operational information to food companies [4,6].

Briefly, summarizing the main findings of previous research, the behavioural response of *S. zeamais* adults to different types of cereal pasta (barley, buckwheat, durum wheat, five cereals, kamut, corn, rice, and spelt) was analysed by Trematerra (2009) [4] in multichoice olfactometric tests. In this research, corn pasta was more attractive than the other tested pasta types; barley, kamut, spelt, and the five cereals pastas were the less attractive to maize weevil adults.

Recently, using as a test insect the congeneric *S. granarius*, Trematerra et al. (2021) [6] compared commercially available Italian macaroni pastas made of different cereals (six different pasta types) or legumes (four pastas made of 100% *Cicer arietinum*; 100% *Lens culinaris*; 100% *Pisum sativum*; and 100% *Vicia faba*). In the multiple-choice test, they found that wheat weevil adults were statistically more attracted to cereal pastas than legume pastas, although in some cases not in a statistically significant manner.

Trematerra et al. (2021) [6] performed a GC-MS analysis of the HS-SPME extracts of the different pasta types and found differences in terms of quantity and quality, with aliphatic aldehydes and aliphatic alcohols as the most abundant volatile components in cereal and legume pulps, respectively. Analysing the response indices in the two-choice behavioural biological tests, the authors suggested that the low attractiveness of legume pasta was mainly due to the lack of attractants rather than the emission of repellent compounds.

The definition of the biological activity of the volatile components in legume-based pastas provides interesting implications from a practical point of view. The identification of any repellent, deterrent, and toxic compounds could, for example, lead to the preparation of bioactive packaging capable of limiting the risk of infestation of packaged products.

In the present experiments with five different pasta types, *S. zeamais* showed a clear preference for pasta made of 100% *T. durum* flour compared to the legume- and legume-enriched pastas. In the multichoice olfactometer bioassays, the mean percentage of insects attracted by the trap devices baited with 100% *T. durum* pasta (61.4%) was significantly higher than those of insects attracted by 100% *L. esculenta* pasta (2.7%), 100% *C. arietinum* pasta (3.8%), 50% *T. durum* + 50% *L. esculenta* pasta (4.2%), and 60% *T. durum* + 40% *C. arietinum* pasta (4.3%). 

In the two-choice behavioural bioassays comparing each pasta type with an empty trap device, positive and significant RIs were elicited by the *T. durum* pasta and by pastas made of *T. durum* + *C. arietinum*, and *T. durum* + *L. esculenta,* indicating actual maize weevil attraction. However, the RI calculated for the *T. durum* pasta was significantly higher than those of the other two attractant pastas, indicating a reduction in attractiveness induced by combinations of *T. durum* with *C. arietinum* or *L. esculenta* flours. Accordingly, the RIs of adult maize weevils to *C. arietinum* or *L. esculenta* pastas were not significant suggesting a neutral effect on insect orientation.

The lower attractiveness of pastas obtained by combining *T. durum* with *C. arietimun* or *L. esculenta* flour compared to pasta made of *T. durum* only confirms the importance of the ratio and concentrations of specific host volatile compounds in insect orientation towards suitable food and oviposition substrates [17,18,19] and provide useful practical information for the safer prevention of maize weevil pasta infestations.

## 5. Conclusions

On the whole, the results of this study clearly demonstrate the great capability of the maize weevil sensorial system to differentiate between various sources of olfactory stimuli. 

The capability of maize weevil males and females to perceive a wide range of volatiles identified from different cereal grains has been demonstrated by electroantennography with 2-hexanone, 2-heptanone, 1-butanol, 3-methyl-1-butanol, 1-hexenol in males, and 1-pentanol, 1-butanol, 3-methyl-1-butanol, 2-heptanone in females, being the strongest antennal stimulants [20]. However, to elucidate the chemical bases of *S. zeamais* attraction to *T. durum* pasta further, chemical, electrophysiological, and behavioural investigations are needed.

The proposal of new types of pasta is increasingly meeting the preferences of the market and some social groups of consumers, including the mixture *T. durum* and legumes. In our case, it is also interesting to note how this simultaneously decreases the risks of infestation by *S. zeamais* adults, a very dangerous and widespread species.

## Figures and Tables

**Table 1:** Nutrition facts of the five different types of commercial macaroni pasta used in the tests.

Samples	Pasta Type Cereal/Legume	Energy Value	Fats g/100 g	Carbohydrates g/100 g	Fibers g/100 g	Proteins g/100 g	Salt g/100 g
A	100% *Triticum durum*	1521 kJ 359 kcal	2.0 0.4 sat.	69.7 3.5 sugar	3.0	14.0	0.013
B	100% *Cicer arietinum*	1465 kJ 348 kcal	6.2 1.1 sat.	45.1 2.9 sugar	14.0	21.0	0.012
C	100% *Lens esculenta*	1401 kJ 331 kcal	1.2 0.3 sat.	49.6 2.0 sugar	11.0	25.0	0.003
D	50% *Triticum durum* + 50% *Cicer arietinum*	1457 kJ 347 kcal	3.9 0.7 sat.	56 1.9 sugar	10	17	0
E	60% *Triticum durum +* 40% *Lens esculenta*	1436 kJ 342 kcal	2.0 0.4 sat.	61 2.5 sugar	6.2	17	0

**Table 2:** Behavioural responses of *S. zeamais* adults in two-choice bioassay. In a row, significant differences between the first and the unbaited control choices are indicated by Student’s *t*-test.

Samples	First vs. Unbaited Control	First Choice (±SE)	Unbaited Control (±SE)	Student’s *t*-Test		Response Index (±SE)
				*t*-Value	*p*-Value	
A vs. F	Pasta made with 100% *Triticum durum* vs. control	79.6 ± 3.7	3.0 ± 0.6	20.285	<0.001	76.6 ± 4.3 a
B vs. F	Pasta made with 100% *Cicer arietinum* vs. control	22.6 ± 2.7	16.8 ± 2.1	1.688	0.130	5.8 ± 2.2 c
C vs. F	Pasta made with 100% *Lens esculenta* vs. control	18.6 ± 3.8	16.2 ± 3.6	0.459	0.658	2.4 ± 3.2 c
D vs. F	Pasta made with 50% *Triticum durum* + 50% *Cicer arietinum* vs. control	37.0 ± 6.5	8.4 ± 2.0	4.187	0.003	28.6 ± 7.7 b
E vs. F	Pasta made with 60% *Triticum durum +* 40% *Lens esculenta* vs. control	25.6 ± 2.5	14.0 ± 2.8	3.127	0.014	11.6 ± 1.1 bc

Within the Response Index column, means followed by the same letter are not significantly different (Tukey HSD post hoc test, *p* < 0.05).

## Data Availability

Data are contained within the article.
